# Impacts of Mist Spray on Rice Field Micrometeorology and Rice Yield under Heat Stress Condition

**DOI:** 10.1038/s41598-020-58578-3

**Published:** 2020-01-31

**Authors:** Xiaodong Jiang, Mengfei Hua, Xiaoya Yang, Ning Hu, Rangjian Qiu, Shenbin Yang

**Affiliations:** Collaborative Innovation Center on Forecast and Evaluation of Meteorological Disasters (CIC-FEMD)/Jiangsu Key Laboratory of Agricultural Meteorology/School of Applied Meteorology, Nanjing University of Information Science & Technology, Nanjing, Jiangsu 210044 China

**Keywords:** Climate sciences, Environmental sciences

## Abstract

Heat stress is one of the common agrometeorological hazards in rice production in the middle and lower reaches of the Yangtze River in China. To study the mechanism of mist spray in ameliorating heat stress injury, a field experiment was conducted at Nanjing (China) with an early and a late hybrid rice varieties (*Oryza sativa* L.). The mist spray treatments were conducted at the flowering period, which were at August 6-10 for early rice variety and September 1-5 for late one. Four treatments at different irrigation times (T1: 08:00; T2: 12:00; T3: 14:00; CK: no mist spray; mist spray amount of 1 L·m^−2^) were included. The temperature and humidity at the different heights of the rice canopy and the net solar radiation above the canopy were measured. The leaf senescence, chlorophyll content, photosynthetic rate and the yields of the rice were determined. The results showed that mist spray rapidly reduced the temperature and increased the relative humidity in the canopy. The cooling effect was most significant at the top of the canopy and decreased downward from the top of canopy. The duration of the temperature decrease caused by the mist spray was 2 h. Mist spray could lead to an increase in latent heat flux (*LE*) and a decrease in sensible heat flux (*H*) in the rice field. The mist spray treatments delayed the senescence of the rice leaves, increased the activity levels of the superoxide dismutase, peroxidase, catalase, and soluble protein, reduced the malondialdehyde content, increased leaf chlorophyll content, photosynthetic rate and yield. The T2 treatment showed the most significant effect against heat stress, with the yield of the two varieties increased 13.7 and 13.6% respectively. Compared with mist spray at 08:00 or 14:00, spraying at 12:00 had the strongest resistance to heat stress in rice field.

## Introduction

The greenhouse effect caused by the development of industry and by greenhouse gas emissions has led to an increase in global temperatures^1,2^. The global surface temperature rose by 0.85 °C between 1880 and 2012^3^. The global temperature is expected to continue increasing at a rate of 0.1 to 0.2 °C/10a in the coming decades^4^, and the temperature will rise by approximately 4.8 °C by 2100^3^. In the context of global warming, the frequency and intensity of extremely high temperature events have increased^5,6^, which will have a significant impact on agriculture^7–9^.

Rice (*Oryza sativa* L.) is one of the most important food crops in the world^10,11^. China is the largest producer and consumer of rice in the world^12^. Heat stress is one of the main agrometeorological disasters in China’s rice production^13,14^ and shows an increasing trend in China’s major rice production areas during 1960–2009^14^. In China, the heat stress of rice is usually defined as more than 3 days in succession with a daily average temperature of ≥30 °C or with a daily maximum temperature of ≥35 °C during the booting - flowering periods of rice^15^. Rice is highly susceptible to heat stress during the reproductive growth period^9,16^, and the flowering stage is the most vulnerable period for rice^17–19^. Studies have shown that heat stress can lead to premature aging of rice, reduced activity of antioxidant enzymes^20–22^, and inactivation of Rubisco; it can also affect the ability of the rice to assimilate CO_2_^23^, impair the structure and functions of photosystem II (PSII), reduce the light energy conversion efficiency of PSII^24^, and cause stomatal closure^25^, thereby resulting in decreased photosynthetic capacity^20,26^. When rice is exposed to high temperature stress during the flowering time, the number of formed pollens and the pollen vigor are reduced, which results in a reduction in the rice seed setting rate, thereby ultimately affecting the rice yield^17,27,28^.

Mist spray is an effective means for alleviating heat stress. When heat stress occurs, mist spray can increase the atmospheric relative humidity within the crop canopy, reduce the water pressure difference, and lower the canopy temperature, thereby significantly affecting the microclimate in the field^29–31^. Changes in the microclimate also cause changes in the physiological characteristics of crops. Mist spray can reduce the crop transpiration rate^30,32^, increase the crop photosynthesis^33,34^, reduce the temperature at the panicles, and increase the pollen viability and fertilization capacity^35,36^. A study by Kong *et al*.^37^ showed that mist spray during the early stage of grain filling in rice where heat stress has occurred could reduce the canopy temperature, reduce the degree of peroxidation of the membrane lipids in the rice leaves, and increase the leaf photosynthesis and rice yield.

Energy balance in the plant canopy is a basic physical process where the soil, vegetation, and atmosphere undergo coupling. The distribution characteristics of each component in the energy balance equation can provide a reliable theoretical basis for the heat flow in rice fields. Although relevant reports exist on the microclimate effect and the physiological response of crops after mist spray, elucidation of the mechanism of mist spray against high temperature is still needed from the perspective of energy balance. The Yangtze River Basin is China’s major rice production area; in this region, rice production accounts for approximately 50% of China’s rice production, and heat stress is the main agrometeorological threat to rice production in this region^14^. In the scenario of future climate change, the occurrence, frequency, and duration/days of heat stress in rice production will continue to increase, which seriously threatens the rice production in this region^6,13,38,39^. For this reason, during the rice flowering stage, when heat stress is likely to occur, mist spray was applied in a rice field at different times to reveal the defense mechanism of mist spray to heat stress in rice from the perspective of energy balance and crop physiology and to find the optimal mist spray time during the occurrence of heat stress, thereby providing a theoretical basis for the cultivation and management measures required for high-yield and stable-yield rice production.

## Results

### Effects of mist spray on canopy temperature

**Figure 1:**
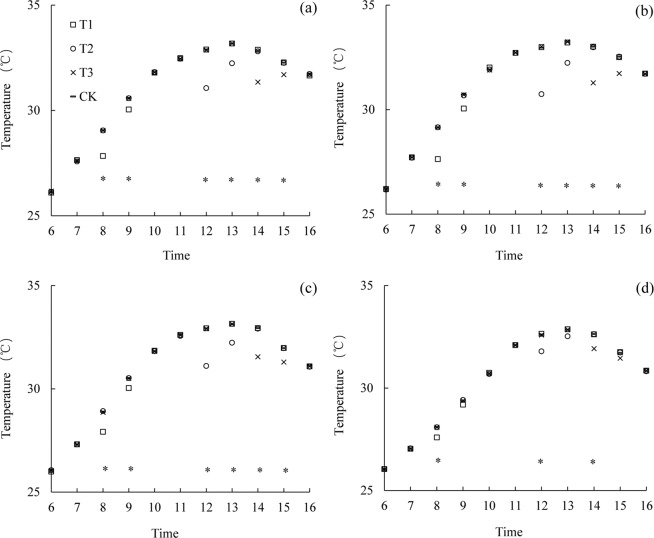
Average diurnal variation of temperature in the canopy at different heights (**a**: 85 cm; **b**: 75 cm; **c**: 50 cm; **d**: 25 cm) for variety of Lingliangyou 268 (LLY 268). *Indicate significant difference among treatments (P < 0.05). T1 is mist spray at 08:00, T2 is mist spray at 12:00, T3 is mist spray at 14:00, and CK is no mist spray. Mist spray was conducted with an electric sprayer, and the spray volume was 1 L/m^2^.

The diurnal variation of temperature at the different canopy heights in the early rice variety LLY 268 under various treatments were shown in Fig. 1. Under all the treatments, the diurnal variation had a bimodal curve change, with the temperature decreasing somewhat after these mist spray treatments. The cooling effect gradually decreased downward from the top of the canopy, and the cooling effect caused by the mist spray lasted for 2 hours. No significant difference existed in the canopy temperature among the various treatments during the no treatment period. Compared with the CK treatment, at the height of 10 cm above the canopy (85 cm), the temperature decreased by 0.85 °C during 8:00 to 9:00 under the T1 treatment; the temperature decreased by 1.35 °C during 12:00 to 13:00 under the T2 treatment; and the temperature decreased by 1.05 °C during 14:00 to 15:00 under the T3 treatment. At the top of the canopy (75 cm), the temperature decreased by 1.1, 1.6 and 1.25 °C under the T1, T2 and T3 treatment respectively. At 2/3 of the plant height (50 cm), the temperature decreased by 0.75, 1.35 and 1.05 °C under the T1, T2 and T3 treatment respectively. At 1/3 of the plant height (25 cm), the temperature decreased by 0.35, 0.55 and 0.5 °C under the T1, T2 and T3 treatment respectively. The T2 treatment exhibited the highest temperature reduction effect compared with T1, T3 and CK.

**Figure 2:**
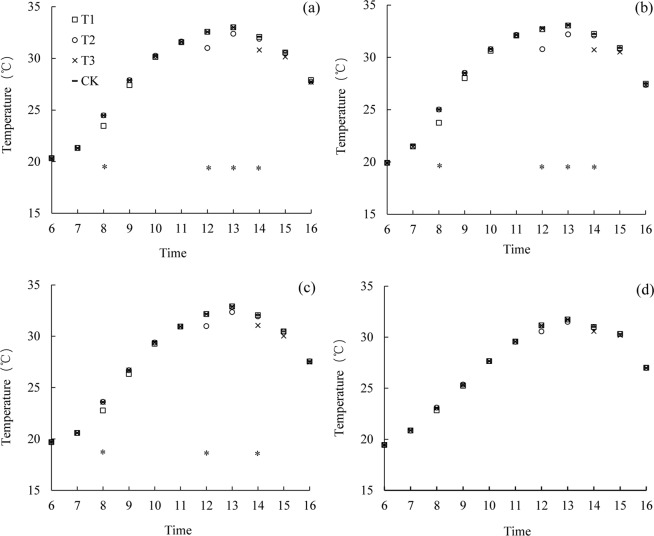
Average diurnal variation of temperature in the canopy at different heights (**a**: 110 cm; **b**: 99 cm; **c**: 66 cm; **d**: 33 cm) for variety of Liangyou Peijiu (LYPJ). *indicate significant difference among treatments (P < 0.05). T1 is mist spray at 08:00, T2 is mist spray at 12:00, T3 is mist spray at 14:00, and CK is no mist spray. Mist spray was conducted with an electric sprayer, and the spray volume was 1 L/m^2^.

The diurnal variation of temperature at the different canopy heights in the late rice variety LYPJ under the various treatments were similar with variety LLPJ (Fig. 2). The cooling effect caused by the mist spray lasted for 2 hours. The cooling effect of T2 was the best, and the second one was T3. Compared with the CK treatment, the canopy temperature of T2 averagely decreased by 1.1, 1.45, 0.9 and 0.4 °C at the height of 10 cm above the canopy (110 cm), the top of the canopy (99 cm), 2/3 of the plant height (66 cm), and 1/3 of the plant height (33 cm) respectively. The temperature reductions of T2 treatment at the four heights were higher than that of T1 treatment by 0.35, 0.55, 0.3 and 0.25 °C respectively, and which were higher than T3 treatment by 0.25, 0.5, 0.2 and 0.15 °C respectively.

### Effects of mist spray on the relative humidity of the canopy

**Figure 3:**
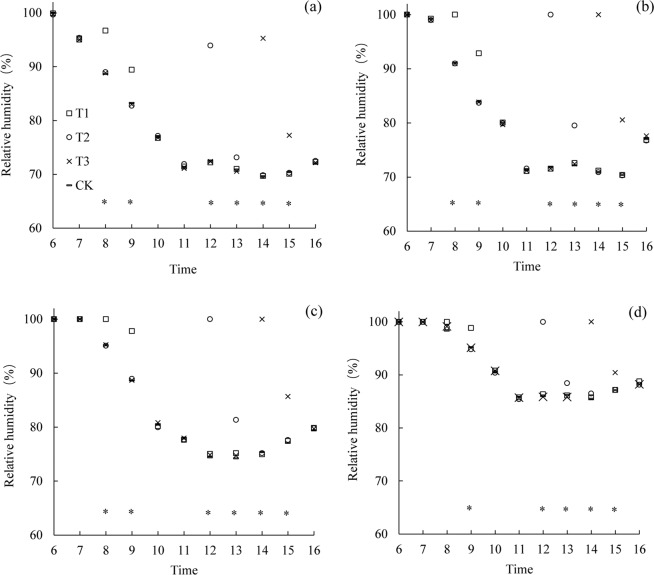
Average diurnal variation of relative humidity in the canopy at different heights (**a**: 85 cm; **b**: 75 cm; **c**: 50 cm; **d**: 25 cm) for variety of Lingliangyou 268 (LLY 268). *indicate significant difference among treatments (P < 0.05). T1 is mist spray at 08:00, T2 is mist spray at 12:00, T3 is mist spray at 14:00, and CK is no mist spray. Mist spray was conducted with an electric sprayer, and the spray volume was 1 L/m^2^.

The diurnal variation of the relative humidity at the different canopy heights in the early rice variety LLY 268 under various treatments were shown in Fig. 3. Under all the treatments, the diurnal variation had a unimodal curve change, with the relative humidity increasing to varying degrees after these mist spray treatments. The increase in relative humidity after the mist spray lasted for 2 h, and no significant difference was observed in the relative humidity of the canopy among the various treatments during the no treatment period. Under all the irrigation treatments, the relative humidity of the canopy increased to 100%. At 1 hour after mist spray, at the height of 10 cm above the canopy (85 cm), the relative humidity increased by 6.2, 2.2 and 6.9% under the T1, T2 and T3 treatment respectively compared with the CK treatment. At the top of the canopy (75 cm), the relative humidity increased by 8.9, 7.4 and 10.2% under the T1, T2 and T3 treatment respectively. At 2/3 of the plant height (50 cm), the relative humidity increased by 9.2, 7.1 and 8.6% under the T1, T2 and T3 treatment respectively. At 1/3 of the plant height (25 cm), the relative humidity increased by 3.6, 2.2 and 3.3% under the T1, T2 and T3 treatment respectively.

**Figure 4:**
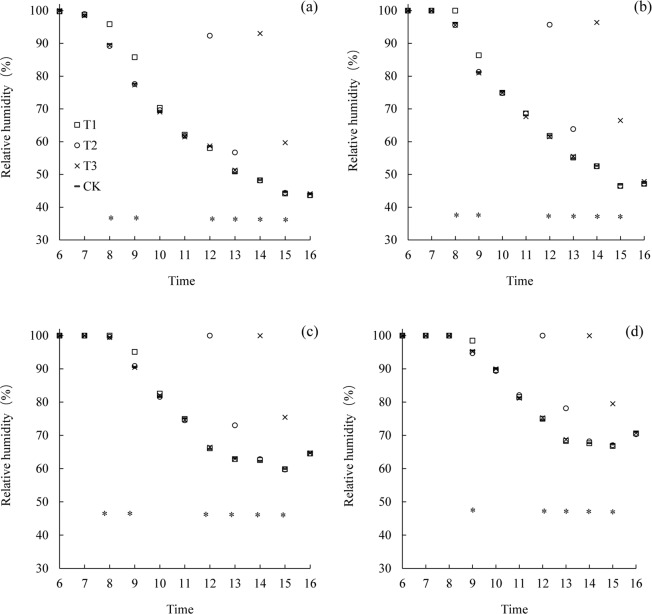
Average diurnal variation of relative humidity in the canopy at different heights (**a**: 110 cm; **b**: 99 cm; **c**: 66 cm; **d**: 33 cm) for variety of Liangyou Peijiu (LYPJ). *indicate significant difference among treatments (P < 0.05). T1 is mist spray at 08:00, T2 is mist spray at 12:00, T3 is mist spray at 14:00, and CK is no mist spray. Mist spray was conducted with an electric sprayer, and the spray volume was 1 L/m^2^.

The diurnal variation of relative humidity at the different canopy heights in the late rice variety LYPJ under the various treatments were similar with the early rice variety LLY 268 (Fig. 4). At 1 hour after mist spray, at the height of 10 cm above the canopy (110 cm), the relative humidity increased by 8.1, 6.2 and 15.2% under the T1, T2 and T3 treatment respectively compared to the CK treatment. At the top of the canopy (99 cm), the relative humidity increased by 5.1, 9.0 and 19.6% under the T1, T2 and T3 treatment respectively. At 1/3 of the plant height (33 cm), the relative humidity increased by 3.0, 10.4 and 12.2% under the T1, T2 and T3 treatment respectively.

### Effects of mist spray on the energy balance components

**Figure 5:**
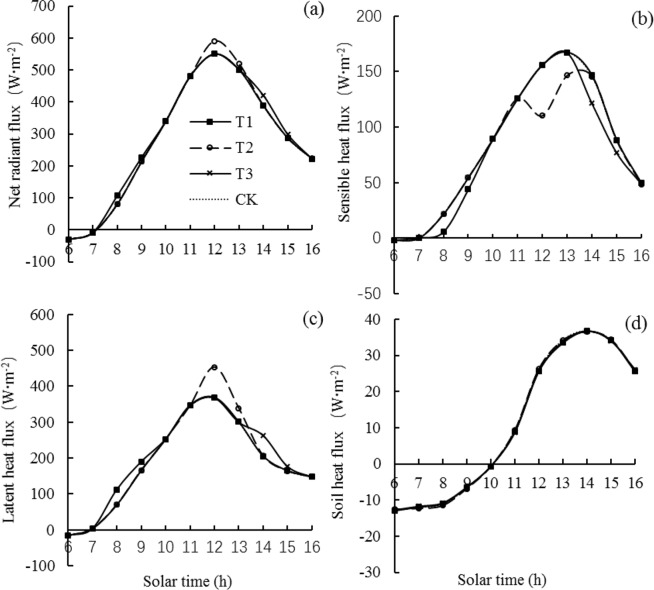
Average diurnal variation in the energy balance components for variety of Lingliangyou 268 (LLY 268). T1 is mist spray at 08:00, T2 is mist spray at 12:00, T3 is mist spray at 14:00, and CK is no mist spray. Mist spray was conducted with an electric sprayer, and the spray volume was 1 L/m^2^.

The diurnal variation in the energy balance components for LLY 268 under different treatments were shown in Fig. 5. The mist spray had no apparently effect on the soil heat flux (*G*) (Fig. 5d), but it changed the net radiant flux (*R*_*n*_), the latent heat flux (*LE*), and the sensible heat flux (*H*) (Fig. 5a–c).

*R*_*n*_ increased under the mist spray at the different times in the rice field (Fig. 5a). Under the T1 treatment, *R*_*n*_ increased in the period from 08:00 to 09:00, with averagely increased amount was 19.49 W·m^−2^ compared with the CK treatment. Under the T2 treatment, *R*_*n*_ increased in the period from 12:00 to 13:00 with averagely increased amount was 28.84 W·m^−2^ compared with the CK treatment. Under the T3 treatment, *R*_*n*_ increased in the period from 14:00 to15:00 with averagely increased amount was 22.09 W·m^−2^ compared with the CK treatment

Different mist spray treatments reduced *H* in the rice field (Fig. 5b). Under the T1 treatment, *H* decreased in the period from 08:00 to 09:00 with averagely decreased amount was 13.55 W·m^−2^ compared with the CK treatment. Under the T2 treatment, *H* decreased at 12:00-13:00 with averagely decreased amount was 33.11 W·m^−2^ compared with the CK treatment. Under the T3 treatment, *H* decreased in the period from 14:00 to 15:00, with an average decrease of 17.80 W·m^−2^ compared with the CK treatment.

Mist spray at different times increased *LE* in the rice field (Fig. 5c). Under the T1 treatment, *LE* increased apparently in the period from 08:00 to 09:00 with an average increase of 32.97 W·m^−2^ compared with the CK treatment. Under the T2 treatment, *LE* increased in the period from 12:00 to 13:00 with an average increase of 61.83 W·m^−2^. Under the T3 treatment, *LE* increased in the period from 14:00 to 15:00 with an average increase of 34.69 W·m^−2^.

**Figure 6:**
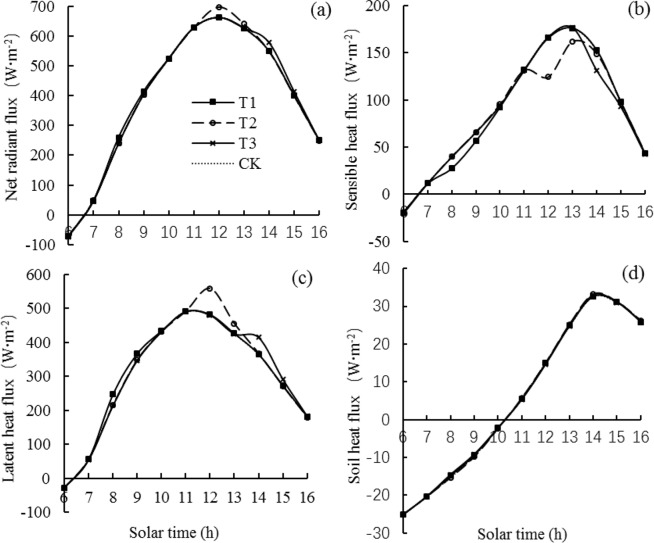
Average diurnal variation in the energy balance components for variety of Liangyou Peijiu (LYPJ). T1 is mist spray at 08:00, T2 is mist spray at 12:00, T3 is mist spray at 14:00, and CK is no mist spray. Mist spray was conducted with an electric sprayer, and the spray volume was 1 L/m^2^.

The diurnal variation in the energy balance components for LYPJ under different treatments were similar with LLPJ (Fig. 6). The mist spray at different times increased *R*_*n*_ in the rice field (Fig. 6a). *R*_*n*_ averagely increased by 14.98, 25.17 and 22.50 W·m^−2^ in the period from 08:00 to 09:00, from 12:00 to 13:00 and from 14:00 to 15:00 respectively compared to the CK treatment. Different mist spray reduced the *H* in the rice field (Fig. 6b). *H* averagely decreased by 10.77, 28.63 and 12.92 W·m^−2^ in the period from 08:00 to 09:00, from 12:00 to 13:00 and from 14:00 to 15:00 respectively compared to the CK treatment. Mist spray at different times increased the *LE* in the rice field (Fig. 6c). *LE* averagely decreased by 25.41, 53.58 and 35.17 W·m^−2^ in the period from 08:00 to 09:00, from 12:00 to 13:00 and from 14:00 to 15:00 respectively compared to the CK treatment.

Different mist spray treatments changed the ratios of *LE/R*_*n*_ and *H/R*_*n*_ in the rice field (Table 1). For LLY 268, the ratio of *LE/R*_*n*_ of T1, T2, T3 treatments were 2.11, 4.37 and 2.11% higher than the CK treatment respectively. For LYPJ, the ratio of *LE/R*_*n*_ of T1, T2, T3 treatments were 0.92, 2.11 and 1.05% higher than the CK treatment respectively. In contrast to *LE*, mist spray reduced the ratio of *H/R*_*n*_. For LLY 268, the ratio of *H/R*_*n*_ of T1, T2, T3 treatments were 4.05, 9.46 and 5.41% lower than the CK treatment respectively. For LYPJ, the ratio of *H/R*_*n*_ of T1, T2, T3 treatments were 3.54, 7.52 and 3.98% lower than the CK treatment respectively. These results showed that the higher the ambient temperature, the faster the water evaporation after spraying, the larger the *LE* increased, the larger the ratio of *LE/R*, the more H decreased, and the smaller the ratio of *H/R*.

**Table 1:** Average diurnal ratio of the various energy components accounting for net radiation.

Variety	Treatment	*H*/*R*_*n*_(%)	*LE*/*R*_*n*_(%)
Lingliangyou 268 (LLY 268)	T1	28.4	67.7
	T2	26.8	69.2
	T3	28.0	67.7
	CK	29.6	66.3
Liangyou Peijiu (LYPJ)	T1	21.8	76.7
	T2	20.9	77.6
	T3	21.7	76.8
	CK	22.6	76.0

*R*_*n*_ is the net radiation received by the rice canopy, *H* is the sensible heat exchange between the rice canopy and air, *LE* is the latent heat exchange between the rice canopy and the air.

T1 is mist spray at 08:00, T2 is mist spray at 12:00, T3 is mist spray at 14:00, and CK is no mist spray. Mist spray was conducted with an electric sprayer, and the spray volume was 1 L/m^2^.

### Effects of mist spray on leaf senescence characteristics

**Figure 7:**
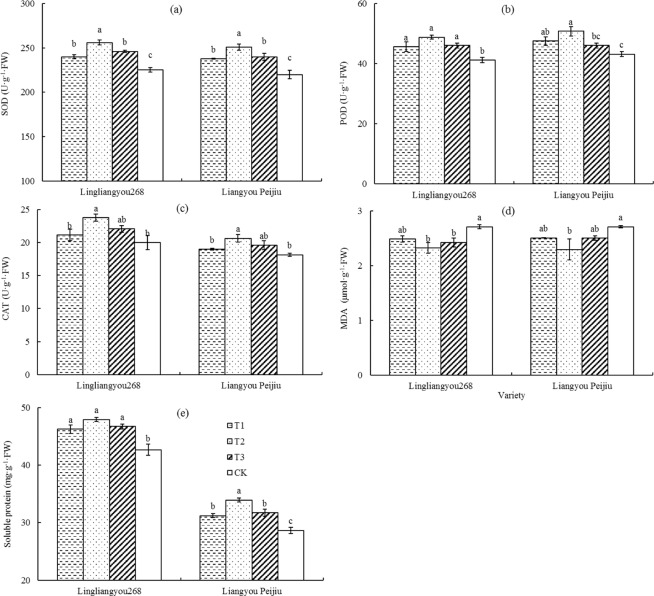
Effect of mist spray at different times on leaf senescence characteristics. T1 is mist spray at 08:00, T2 is mist spray at 12:00, T3 is mist spray at 14:00, and CK is no mist spray. Mist spray was conducted with an electric sprayer, and the spray volume was 1 L/m^2^. Error bars represent standard errors of the means. Different letters above error bars indicate significant difference among treatments by LSD test (P < 0.05).

Antioxidant enzymes (SOD, POD, and CAT) in plants can inhibit the oxidation in plants and thus have a role in delaying plant senescence. However, with the aging process in plants, the antioxidant enzyme activity and the soluble protein content in the plants also decrease, and the MDA content increases. The effects of mist spray on leaf senescence characteristics in rice were shown in Fig. 7, which showed that the SOD activity, POD activity, CAT activity, MDA content, and soluble protein content were changed after mist spray. Specifically, the activity levels of the SOD, POD, and CAT and the soluble protein content increased, and the MDA content decreased.

The effects of mist spray at different times on the SOD activity were shown in Fig. 7a. The SOD activity increased by 29.37, 61.47 and 41.30 U·g^−1^·FW under the T1, T2 and T3 treatment respectively for LLY 268 compared with the CK treatment. The SOD activity increased by 35.68, 61.62 and 39.66 U·g^−1^·FW under the T1, T2 and T3 treatment respectively for LYPJ compared with the CK treatment. The POD activity increased by 4.47, 7.59 and 4.83 U·g^−1^·FW under the T1, T2 and T3 treatment respectively for LLY 268, which increased by 4.37, 7.59 and 2.83 U·g^−1^·FW under the T1, T2 and T3 treatment respectively for LYPJ (Fig. 7b). The CAT activity increased by 1.15, 3.80 and 2.08 U·g^−1^·FW under the T1, T2 and T3 treatment respectively for LLY 268compared with the CK treatment, and which increased by 0.85, 2.48 and 1.42 U·g^−1^·FW under the T1, T2 and T3 treatment respectively for LYPJ (Fig. 7c). The MDA content decreased by 0.22, 0.39 and 0.29 μmol·g^−1^·FW under the T1, T2 and T3 treatment respectively for LLY 268 compared with the CK treatment, and which decreased by 0.20, 0.42 and 0.21 μmol·g^−1^·FW under the T1, T2 and T3 treatment respectively for LYPJ (Fig. 7d). The soluble protein content increased by 3.57, 5.26 and 4.05 mg·g^−1^·FW under the T1, T2 and T3 treatment respectively for LLY 268 compared with the CK treatment, which increased by 2.57, 5.27 and 3.06 mg·g^−1^·FW under the T1, T2 and T3 treatment respectively for LYPJ (Fig. 7e).

In summary, compared with T1, T3 and CK, T2 treatment had the highest SOD, POD, and CAT activity levels; the highest soluble protein content; and the lowest MDA content, thereby showing the lowest degree of leaf senescence.

### Effects of mist spray on leaf chlorophyll content and photosynthetic rate

**Figure 8:**
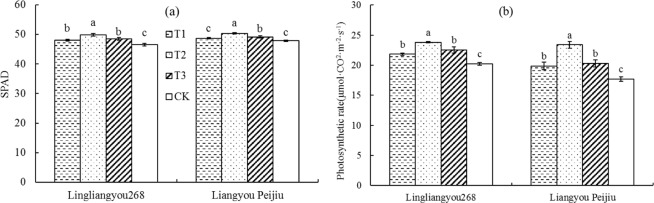
Effect of mist spray at different times on leaf chlorophyll content and photosynthetic rate. T1 is mist spray at 08:00, T2 is mist spray at 12:00, T3 is mist spray at 14:00, and CK is no mist spray. The spray volume was 1 L/m^2^. Error bars represent standard errors of the means. Different letters above error bars indicate significant difference among treatments by LSD test (P < 0.05).

Different spraying treatments also affected the chlorophyll content and photosynthetic rate of flag leaves of rice. Chlorophyll content of rice flag leaf is an important index of photosynthetic capability. Compared with CK, the chlorophyll content of flag leaves were significantly increased by different treatments (Fig. 8a), but the increasing rate was different. The increasing rate of chlorophyll content of flag leaves under Treatment T1, T2 and T3 were 3.29%, 7.25%, 4.15% in LLY 268 and 1.82%, 5.38%, 2.78% in LYPJ respectively. Figure 8b shows the effect of different treatments on photosynthetic rate of rice flag leaf. Spraying treatment could increase the photosynthetic rate of flag leaves significantly. The photosynthetic rates of flag leaves with T1, T2 and T3 were 1.60, 3.60, 2.30 μmol·CO_2_·m^−2^·s^−1^ higher than CK in LLY 268, 2.16, 5.68, 2.58 μmol·CO_2_·m^−2^·s^−1^ higher than CK in LYPJ, respectively.

### Effects of mist spray on rice yield components

All the different mist spray treatments increased the yield of rice (Table 2). The yield increased by 7.1, 13.7 and 8.2% under the T1, T2 and T3 treatment respectively for LLY 268 compared with the CK treatment. The yield increased by 4.1, 13.6 and 6.0% under the T1, T2 and T3 treatment respectively for LYPJ. According to the analysis of the yield components under the different treatments, the different treatments had no significant effect on the number of panicles per unit area of rice field and the thousand-grain weight, but the treatments could increase the spikelet number per panicle and the seed setting rate somewhat, thereby causing a yield increase. The spikelet number per panicle under the T2 treatment was 5.78 spikelets/panicle higher than the CK treatment for LLY 268; no significant differences were observed between the T1 and the CK or between the T2 and the CK. For LYPJ, the spikelet numbers per panicle increased by 3.66, 9.66 and 5.00 spikelets·panicle^−1^ under the T1, T2, and T3 treatments respectively compared with the CK. The seed setting rate increased by 5.4, 8.5 and 5.8% under the T1, T2, and T3 treatment respectively compared with the CK for LLY 268. For LYPJ, the seed setting rate increased by 3.3, 6.9 and 4.5% under the T1, T2, and T3 treatment respectively. Under the different mist spray treatments, the increase in yield was mainly due to the increase in the seed setting rate, followed by the increase in the spikelet number per panicle; both factors showed a significant increase under the T2 treatment compared with T1, T3 and CK, and accordingly, the yield increase effect was the most significant under this treatment.

**Table 2:** Rice yield and its components in different treatments.

Variety	Treatment	Number of panicles (panicles/m^2^)	Thousand-grain weight (g/Thousand-grain)	Spikelet number (Spikelets/panicle)	Seed setting rate (%)	Yield (g/m^2^)
Lingliangyou 268 (LLY 268)	T1	242.67	23.23	131.78	82.30	749.60
	T2	242.33	23.47	136.11	84.74	796.07
	T3	239.67	23.49	132.89	82.61	757.30
	CK	243.67	23.20	130.33	78.09	699.87
Liangyou Peijiu (LYPJ)	T1	211.33	26.14	201.22	81.83	834.09
	T2	213.33	26.43	207.22	84.72	909.38
	T3	208.45	26.24	202.56	82.83	848.27
	CK	210.33	26.28	197.56	79.25	800.62

Within a column, values in the same variety followed by different letters are significantly different among treatments by LSD test (P < 0.05).

T1 is mist spray at 08:00, T2 is mist spray at 12:00, T3 is mist spray at 14:00, and CK is no mist spray. Mist spray was conducted with an electric sprayer, and the spray volume was 1 L/m^2^.

## Discussion

The flowering stage is a critical period for the establishment of rice yield organs, and it is also the period most affected by heat stress^17–19,24^ as well as being the period where defending against heat stress is most effective. The results of this experiment indicate that mist spray in a rice field at different times could reduce the canopy temperature in the rice field somewhat, to effectively relieve the influence of high temperature on the growth of rice, thereby increasing the yield.

Relative humidity plays an important role when dealing with heat stressWeerakoon *et al*.^40^ and Yan *et al*.^41^ documented that the spikelet sterility of rice increased under the condition of high temperature (>30 °C) and high humidity (>80%), while sterility decreased significantly with decreasing relative humidity. Their findings may be the result of decreased transpiration as relative humidity increases and increase the temperature inside spikelet. In this study, mist spray increased the relative humidity of rice canopy, which increased the latent heat flux of rice canopy, reduced the temperature of rice canopy, and thus relieved the heat stress of rice. On the other hand, the high relative humidity condition lasted for up to 2 hours and did not increase the heat stress. As reported by other studies^29–32^, after mist spray, the relative humidity within the rice canopy and the residual moisture in the rice leaves increased, and the evaporation of water was able to reduce the canopy temperature. The results of this study also show that mist spray in the rice field at different times had different cooling effects on the rice canopy. The cooling effect of mist spray had the order of T2 > T3 > T1; that is, the higher the external ambient temperature was, the more effective the cooling effect of the mist spray. Wangs’ study also confirmed this point^42^.

From the opinion of energy balance, mist spray increase the water evaporation in rice fields, mainly affects the canopy temperature by changing the magnitude of the *H* and *LE* components, with latent heat exchange serving as an important approach for energy conversion in rice fields^43^; that is, *LE* and *LE/R*_*n*_ in rice field systems increased after mist spray (Fig. 6 and Table 1). In contrast to *LE*, mist spray reduced *H* and *H/R*_*n*_. This reduction occurred mainly because mist spray reduced the canopy temperature, resulting in a reduction in the temperature gradient between the canopy and the air and a decrease in *H* in the rice field system. *LE/R*_*n*_ of T2 treatment were garter than the other treatments, and *H/R*_*n*_ lower than the other treatments. Under T2 treatment, *LE* showed a large increase, and the cooling effect in the rice field was the most apparent, which caused the greatest reduction rate in the temperature gradient between the canopy and the air, thereby resulting in the greatest increase in *LE/R*_*n*_ and reduction rate in *H/R*_*n*_.

More than 60% of the dry matter accumulation of rice yield comes from the photosynthesis of leaves after flowering^44^. Many studies have shown that heat stress would lead to accelerated leaf senescence and decreased photosynthetic capacity^21–26^. Consistent with the results of another study^37^, the results of this study showed that during heat stress, mist spray significantly increased the SOD, POD, and CAT activity levels in the flag leaves of rice, reduced the MDA content, increased the antisenescence ability of leaves, and improved chlorophyll content and photosynthetic rate of the leaves. A comparison of the effects of mist spray at different times showed that the T2 treatment had the best effect in alleviating heat stress. This effect occurs because mist spray was conducted in the rice field at 12:00 under the T2 treatment, when the maximum temperature of the day was imminent, in comparison to the mist spray conducted at 08:00 (in the morning, prior to the occurrence of high temperature) and at 14:00 (in the afternoon, after the occurrence of high-temperature damage); therefore, the mist spray at 12:00 (T2) was more effective in alleviating the damage of high temperature to rice leaves.

Rice yield is determined by the panicle number per unit area, spikelet number per panicle, seed setting rate and grain weight. Since mist spray treatment in this paper was carried out within 5 days during flowering stage and under heat stress condition, it showed no significant effect of the treatment on panicle number per unit area and grain weight, which can be inferred from the yields shown in Table 2. The effect of different mist spray treatments on rice yield mainly affected the spikelet number per panicle and seed setting rate, of which the seed setting rate changed significantly. Seed setting rate is one of the important components of yield and is sensitive to high^17,20,27,28^. The results in this paper indicated that the difference in rice yield among treatments was largely attributed to the difference in seed setting rate under heat stress conditions. Therefore, with cooling effect, mist spray can raise seed setting rate, increase rice yield.

The active flowering time of rice mainly occurs from 09:00 to 12:00, which lasts for 1-2.5 hours a day^45,46^.Flowering stage is very sensitive to meteorological conditions such as temperature, relative humidity, and so on. High temperature reduces pollen production and pollen viability and causes deterioration in stigma fertility^20,47–49^. Directly exposure to high relative humidity or precipitation would inhibit the flowering of rice and result in water uptake-induced pollen grain rupture and decrease the seed setting rate^17,27^. Mist spray could be an effective way to reduce high temperature during 10:00-12:00, the peak period of rice flowering. However, the cooling effect by mist spray comes with significant rise of relative humidity in the rice field. It is known that higher relative humidity accelerates the rupture of pollen grains and increases the rupture fraction of pollen grains. It also reduces the setting rate of rice. Therefore, in this study, the treatment with mist spray at 10:00 was not included.

The results in this paper showed that mist spray was able to reduce heat stress in rice field. Under the T2 treatment, mist spray was conducted after the peak flowering time of rice and before the daily maximum temperature was approaching. The generated cooling effect not only reduced the damage of rice pollens from heat stress but also helped rice avoid the decrease in the number of flowers and the water uptake-induced pollen rupture, which thereby promoted an increase of seed setting rate and yield. Spray quantity is an important factor to regulate canopy temperature in rice field. In this study, quantitative spray treatments were carried out at different times and lasted for about 15 minutes at each time. During each treatment, high relative humidity conditions in the rice field were produced and not lasted for a long time. However, compared with CK, there were not records of further heat damage were observed after the treatment, except an evident cooling effect. As for the optimum mist spray volume for rice field under heat stress condition and how to control relative humidity, especially in the field condition for avoiding damages by high relative humidity and high temperature it still needs further study and well-designed experiments.

## Conclusions

Mist spray reduced air temperature at different height levels of the canopy. The cooling effect was most significant at the top of the canopy, with a cooling effect from mist spray occurring for 2 hours. Mist spray at different times increased the proportion of latent heat flux in different degrees and reduced the proportion of sensible heat flux. Mist spray increased the SOD, POD, CAT activity levels and the soluble protein content, decreased the MDA content in the leaves; and increased the seed setting rate, which ultimately led to an increase in yield. Mist spray at 12:00 in rice fields enabled the highest resistance to heat stress. Mist spray at 12:00 in rice fields activated the highest resistance to heat stress when compared with the effect of the spray at 08:00 or 14:00.

## Materials and Methods

### Experimental design

This experiment was conducted in 2016 at the Agricultural Meteorological Experiment Station (32°03′N, 118°51′E) of Nanjing University of Information Science & Technology, Nanjing, Jiangsu Province, China. Two tested rice varieties were Lingliangyou 268 (LLY 268, early hybrid rice) and Liangyou Peijiu (LYPJ, late hybrid rice), which have been widely planted in the middle and lower reaches of the Yangtze River. Both rice varieties are intermediate heat resistant type. The sowing date was May 15th, and the rice seedlings were raised by the thin film moistening technique until the seeding age of 30 days. Transplantation occurred on June 15, and harvest occurred on September 14 and October 19, respectively. The transplanting density was 23 hills/m^2^, with one seedling per hill; plant spacing was 17 cm; and the row spacing was 26 cm. The tillage-layer soil in the test plots was loamy clay. The total nitrogen and organic carbon contents were 1.5 g·kg^−1^ and 19.4 g·kg^−1^, respectively, and the pH was 6.25. In the test plots, 750 kg·hm^−2^ NPK (nitrogen, phosphorous, and potassium) compound fertilizer (15-15-15) was applied, of which 60% was used as the base fertilizer, and 40% was applied as topdressing at the jointing stage.

**Figure 9:**
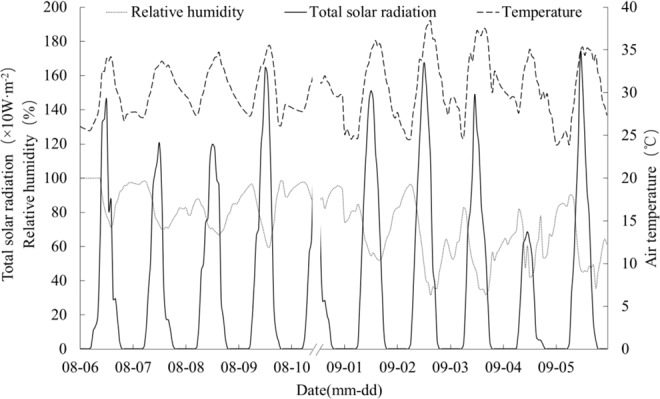
Meteorological data during mist spraying.

The experiment was conducted at August 6-10 (the flowering period of LLY 268) and September 1-5 (the flowering period of LYPJ), when heat stress was occurring in the rice (Fig. 8). Four treatments were included in this experiment: T1 indicates mist spray at 08:00, T2 indicates mist spray at 12:00, T3 indicates mist spray at 14:00, and CK indicates no mist spray. Mist spraying was carried out using an electric sprayer with centrifugal nozzle, where the nozzle diameter is 0.5 mm. Water was sprayed into air around plants to form fog which covered the canopy evenly, and the spray volume was 1 L/m^2^. Three times were included for each treatment, and the area of each plot was 5 m × 5 m. Mist spray lasted about 15 min. During the experiment, the temperature and relative humidity were recorded by a temperature and humidity recorder (HOBO U23-001, Onset, USA). The observed heights included height of 1/3 of the rice plant (25 cm for LLY 268 and 33 cm for LYPJ), height of 2/3 of the rice plant (50 cm for LLY 268 and 66 cm for LYPJ), height at the top of the rice plant (75 cm for LLY 268 and 99 cm for LYPJ), and height 10 cm above the rice plants (85 cm for LLY 268 and 110 cm for LYPJ). The radiation above the canopy was acquired by a four-component net radiation sensor (CNR4, Kipp & Zonen, NED), with an observation height of 150 cm. The soil heat flux (−5.0 cm) was obtained with a heat flux plate (HFT03, Campbell Scientific, USA). The observation data were automatically collected by a datalogger (CR3000, Campbell Scientific Inc., USA), with an acquisition frequency of 1 Hz and with output data recorded as half-hour average values. Meteorological data during the experiment are shown in Fig. 9. From August 6 to 10, the average temperature of each day is 30.05 °C, 30.39 °C, 31.04 °C, 31.39 °C, 30.31 °C, and the duration for excess 35 °C is 0 h, 0 h, 0 h, 4 h, 0 h, respectively. From September 1 to 5, the average temperature of each day is 30.33 °C, 31.45 °C, 31.09 °C, 31.35 °C, 29.65 °C, and the duration for excess 35 °C is 4 h, 6 h, 7 h, 1 h, 3 h, respectively.

### Calculation of energy balance

The energy balance can be calculated from the difference between the energy income and energy expenditure as follows^50^:

1 $${R}_{n}=H+LE+G$$

where *R*_*n*_ (W·m^−2^) is the net radiation received by the rice canopy and directly measured by a net radiation sensor, *LE* (W·m^−2^) is the latent heat exchange between the rice canopy and the air, *H* (W·m^−2^) is the sensible heat exchange between the rice canopy and air, and *G* (W·m^−2^) is the soil heat flux.

Sensible heat flux and latent heat flux can be calculated according to the Bowen ratio-energy balance method^51^:

2 $$\beta =H/LE=\gamma \cdot \varDelta T/\varDelta e$$

where *β* is the Bowen ratio, *γ* is psychrometric constant and is taken as 0.667 hpa/°C, ΔT is the temperature difference between two heights (°C), and Δ*e* is the difference in the vapor pressure between two heights (hPa).

From Eqs. (1) and (2), we have

3 $$H=({{\rm{R}}}_{{\rm{n}}}-G)\cdot \beta /({1}+\beta )$$

4 $$LE=({R}_{n}-G)/({1}+\beta )$$

### Determination of enzyme activity, malondialdehyde content, and soluble protein content

Flag leaves were picked from the rice plant and immediately immersed in liquid nitrogen and stored in a freezing chamber at −40 °C at August 11 (LLY268) and September 6 (LLPJ). Superoxide dismutase (SOD; EC 1.15.1.1) activity was measured by the methods of Rabinowitch and Sklan^52^. It was found out that one unit amount of SOD activity is inhibits at 50% nitro blue tetrazolium (NBT) effect measured at 560 nm per min (UV-1800, Simadzu, Japan). Peroxidase (POD; EC 1.11.1.7) activity was measured using the Chance and Maehley method^53^ whereby POD activity is estimated in rate change in absorbance of reacting solution per unit amount of enzyme at 470 nm every 30 seconds (940 nm per min). Catalase (CAT; EC 1.11.1.6) activity was measured by decomposition reaction of hydrogen peroxide (H_2_O_2_), directly followed by a decrease in absorbance at 240 nm^54^. The enzymatic activity of CAT is equivalent to the rate of decomposition of H_2_O_2_ which is 1μmol per min. The enzymatic activity was measured in enzyme’s unit amount per g of FW. The soluble protein content was determined according to the method of Bradford^55^. The malondialdehyde (MDA) concentration was used to evaluate the lipid peroxidation according Stewart and Bewley^56^. The following formula was applied:

5 $${{\rm{C}}}_{{\rm{MDA}}}({\rm{\mu }}{\rm{mmol}}\cdot {{\rm{g}}}^{-1}{\rm{FW}})=6.45({{\rm{D}}}_{532}-{{\rm{D}}}_{600})-0{{\rm{.56D}}}_{450}\times {\rm{V}}/{\rm{m}}$$

Where 532, 450 and 600 refer to the absorbance wavelength, FW is fresh weight, V is the volume of extracted solution from leaves while m is the mass of sample of leaves.

### Determination of photosynthetic rate and chlorophyll content

Photosynthetic rate and chlorophyll content of flags were measured at August 10 (LLY268) and September 6 (LLPJ). The photosynthetic rate of flag leaves was measured with a portable photosynthetic system (Li-6400, LiCor.Inc., USA) in clear day from 9:30 to 11:30 under the conditions of light intensity of 1000 μmol·m^−2^·s^−1^ and chamber CO_2_ concentration of 390 ± 10 μmol mol^−1^. Each measurement was repeated for 3 times with different plants. The chlorophyll content of flag leaves was measured by a chlorophyll meter (SPAD-502, Minolta Camera Co. Ltd., Japan) and was represented in arbitrary units (SPAD units). Five flag leaves were randomly selected for measurement, and the average value of every five leaves was taken as a repetition. Each measurement was repeated for 3 times with different plants.

### Determination of yield components

In the rice harvest period, 2 m^2^ rice with uniform growth was selected for marking in each test plot. For rice plants within the marked range, the number of effective panicles was counted. After that, the rice was fully harvested. From the harvested rice panicles in each plot, 20 panicles were collected randomly, and the spikelet number per panicle, seed setting rate, and thousand-grain weight were investigated. After being air-dried to a water content of 13 ± 1%, the rice was threshed and weighed.

### Data analysis

SPSS 12.0 was used for data processing and analysis of variance (ANOVA). The least significant method (LSD) was used to perform statistical tests on the data between the different treatments at the 5% level. Excel 2010 was used for graphing. Because the diurnal variation trend of temperature was the same and no sprinkler treatment was performed during the night, the temperature and energy balance components during the 5 days were averaged, and data from the period of 06:00 to 16:00, where differences were exhibited, were extracted and analyzed.
